# Associations between Urinary Excretion of Cadmium and Proteins in a Nonsmoking Population: Renal Toxicity or Normal Physiology?

**DOI:** 10.1289/ehp.1205418

**Published:** 2012-10-31

**Authors:** Magnus Akerstrom, Gerd Sallsten, Thomas Lundh, Lars Barregard

**Affiliations:** 1 Department of Occupational and Environmental Medicine, Sahlgrenska University Hospital and University of Gothenburg, Gothenburg, Sweden; 2 Department of Occupational and Environmental Medicine, Lund University Hospital, Lund, Sweden

**Keywords:** albumin, alpha-1-microglobulin, cadmium, cadmium toxicity, kidney effect, renal function, urinary excretion

## Abstract

Background: Associations between cadmium (Cd) and kidney function have been reported even at low levels of exposure in the general population. Recently, the causality of these associations has been questioned.

Objectives: We examined associations between urinary Cd (U-Cd; a biomarker of exposure) and urinary proteins that are used as biomarkers of kidney effects, based on repeated short-term sampling in healthy subjects.

Methods: Twenty-four hour urine samples were collected on 2 separate days at six fixed times from 30 healthy nonsmoking men and women (median age 39 years). We analyzed the samples (*N* = 354) for Cd (i.e., U-Cd) and two proteins used as kidney function biomarkers: urinary albumin (U-Alb) and alpha-1-microglobulin (U-A1M). Concentrations were adjusted for creatinine concentration or for specific gravity, and excretion rates (mass per hour) were calculated. Possible associations were assessed within each individual participant, and mean correlations and regressions were evaluated.

Results: We found clear positive mean associations within individuals between the excretion of U-Cd [mean, 0.11 µg/g creatinine (range, 0.01–0.52 µg/g creatinine)] and both U-Alb and U-A1M. The associations were stronger for excretion rates and concentrations adjusted for specific gravity than for concentrations adjusted for creatinine. We also found significant positive associations of urinary flow with excretion of U-Cd, U-Alb, and U-A1M.

Conclusions: Associations between short-term changes in U-Cd and markers of kidney function within individual nonsmoking study participants are unlikely to reflect effects of Cd toxicity. A more likely explanation is that these associations result from normal variation in renal function, including changes in urinary flow, that influence the urinary excretion of both Cd and proteins in the same direction. These effects of normal variability may result in overestimation of the adverse effects of Cd on kidney function at low-level Cd exposure.

Adverse effects of cadmium (Cd) on the human kidney have been demonstrated in occupational settings with high Cd exposure since the late 1940s (Friberg 1950). For the general population, diet is the main source of Cd, except among smokers, for whom tobacco smoking is also an important route of exposure. In the last couple of decades, evidence of renal tubular damage, based on increased excretion of low molecular weight (LMW) proteins, has been reported in association with lower levels of Cd exposure in the general population [Åkesson et al. 2005; European Food Safety Authority (EFSA) 2009; Järup and Åkesson 2009; Järup et al. 1998; Nordberg et al. 2007]. Cd excreted in urine (U-Cd) is widely used to assess exposure or body burden of Cd in the general population, and the excretion of LMW proteins such as beta-2-microglobulin is used as a measure of adverse effects on the kidney (EFSA 2009; Järup et al. 1998). Another LMW protein in urine used in evaluating effects on kidney function is alpha-1-microglobulin (U-A1M) (Åkesson et al 2005; Bakoush et al. 2001; Penders and Delanghe 2004). Urinary excretion of high molecular weight (HMW) proteins such as urinary albumin (U-Alb) have also been reported to increase at high Cd exposure (Nordberg et al. 2009). The negative association between diuresis and concentrations of biomarkers in urine is well known; therefore adjustment for dilution based on urine creatinine concentration (U-Crea) or specific gravity (SG) is used to avoid false-positive associations when biomarkers are measured in individual spot urine samples (Berlin et al. 1985; Suwazono et al. 2005; Trevisan et al. 1994). In contrast, when urine is collected over time, excretion rates can be calculated directly.

Recently the causality of associations between U-Cd and biomarkers of kidney effects in populations with low levels of exposure has been questioned because of possible confounding by smoking or physiological sources of variability (Bernard 2008; Chaumont et al. 2010, 2012; Haddam et al. 2011). Several possible physiological mechanisms have been suggested; for example, LMW proteins and Cd-bound metallothionein may have similar affinity for renal tubular binding sites, and excretion of both proteins and U-Cd may be influenced by normal variation in diuresis (i.e., urinary flow rate) (Chaumont et al. 2012). Smoking may be a confounder if it increases the excretion not only of U-Cd but also of urinary proteins (Bernard 2008; Chaumont et al. 2010; Haddam et al. 2011).

The possible influence of physiological factors on associations between U-Cd and biomarkers of kidney function is difficult to reveal in studies using only one spot urine sample per individual participant because it is not possible to disentangle intra- and interindividual variability. Although average excretion of U-Cd and urinary proteins may be relatively stable over time within individuals—and may therefore accurately reflect long-term effects of Cd on kidney function—excretion of both biomarkers may vary over the day because of physiological factors such as diuresis, body position, and exercise. Then these short-term changes would not reflect biological effects of Cd toxicity. Therefore, if there are positive associations between the excretion of U-Cd and urinary proteins over the day within individuals, such associations cannot be assumed to be due to Cd toxicity.

Our aim was to study the association between low-level urinary excretion of Cd and two urinary proteins (U-Alb and U-A1M) within individuals to assess whether associations reported between urinary excretion of Cd and proteins used as biomarkers of impaired kidney function may in fact be due to temporary effects of physiological factors such as a change in urinary flow rate on the biomarkers, rather than long-term toxic effects of Cd on kidney function.

## Materials and Methods

*Study population.* Thirty nonsmoking healthy (i.e., no diabetes, hypertension, or kidney disease) participants (15 men and 15 women) were recruited from the staff of our department and among students at the University of Gothenburg (Gothenburg, Sweden). Each participant filled out a questionnaire concerning age, weight, height, smoking habits, and diseases or medications. Median (range) age and body mass index (BMI) of the participants was 39 years of age (23–59 years) and 23.9 kg/m^2^ (19.1–28.7 kg/m^2^). The study was approved by the ethics committee of the University of Gothenburg and complied with all applicable requirements of international regulations. Participants gave written informed consent prior to the study.

*Urine samples.* Timed samples (*N* = 354) were collected and the volumes recorded on 2 separate days (mostly 4–6 days apart) at six fixed times over 24 hr [at 0930, 1200, 1430, 1730, and 2200 hours and first morning urine (overnight samples)]. If urination was necessary between the fixed times, the next container was used to ensure that all urine was collected over the 24-hr period. Individual sample volumes ranged from 20 to 1,075 mL (median 245 mL), and 24-hr urine volume ranged from 465 to 4,660 mL (median 1,640 mL). No significant sex differences were found. Detailed instructions were given to ensure completeness of the 24-hr sampling. The collected samples were then transferred to Minisorb tubes (NUNC, Roskilde, Denmark) and kept at 4°C until analysis of proteins and U-Crea was performed (within 3 days of collection). Aliquots used for the determination of U-Cd were frozen and analyzed 5 years later. For one woman, samples were collected during 1 day only.

*Chemical analysis.* U-Cd concentrations corrected for molybdenum oxide (MoO) interference were determined at the Department of Occupational and Environmental Medicine, Lund University (Lund, Sweden), by inductively coupled plasma mass spectrometry (Thermo X7; Thermo Elemental, Winsford, UK) in samples diluted 10 times with an alkline solution according to Barany et al. (1997). By adding molybdenum (Mo) to blank urine, the formation of MoO (calculated as Cd) was evaluated. The addition of 500 µg Mo/L contributed an average of 0.68 µg Cd/L. Mo was determined in all samples, thus a correction for the MoO interference was feasible by the known proportion of Mo/MoO. Because the oxidation level could vary from day to day, blank urine with and without added Mo (500 µg Mo/L) was analyzed among the urine samples in every series of analysis. All samples were prepared in duplicate, and the method imprecision (calculated as the coefficient of variation for duplicate preparations) was 9.5%, and for samples with Cd content ranging from the limit of detection (LOD) to 0.10 µg/L, the imprecision was 11%. LOD for U-Cd was 0.05 µg/L, and in 92 of 354 samples (26%), U-Cd was < LOD. Three different quality control samples [Trace Elements Whole Blood (Seronorm AS, Billingstad, Norway) and human blood reference material (Le Centre de Toxicologie du Quebec, International Comparison Program, Quebec, Canada)] were used and the results of testing the three quality control samples versus their stated values (± SD) were 0.26 ± 0.03 µg/L (*n* = 12) versus 0.26–0.36 µg/L, 0.93 ± 0.04 µg/L (*n* = 12) versus 1.01 ± 0.09 µg/L, and 4.9 ± 0.12 µg/L (*n* = 12) versus 5.1 ± 0.26 µg/L, respectively.

Analyses of U-Alb concentrations were performed at the Department of Clinical Chemistry, Sahlgrenska University Hospital (Gothenburg, Sweden) using an automated nephelometric immunochemical method with reagents and a calibrator from Beckman Coulter (Fullerton, CA, USA). Internal reference samples were used in each analytical run, showing satisfactory results. The LOD for U-Alb was 2.4 mg/L, and in 113 of 354 samples (32%), U-Alb was < LOD.

Analyses of U-A1M concentrations were performed at our department using the α_1_-microglobulin ELISA kit K6710 (Immundiagnostik AG, Bensheim, Germany) as described elsewhere (Andersson et al. 2008). Calibrators, which were provided in each kit and had target values ranging from 0.09 to 0.28 mg/L, were always within their acceptable range. The LOD for U-A1M was 0.1 mg/L, and 1 of 354 samples (0.3%) was < LOD.

Analyses of U-Crea concentrations were performed in fresh urine using the Jaffé method (Roche Diagnostics, Mannheim, Germany), with an LOD of 0.01 mmol/L. SG was measured in fresh urine with a Ceti Digit 012 refractometer (Medline, Oxfordshire, UK).

Excretion rates of U-Cd, U-Alb, U-A1M, and U-Crea were calculated from urinary concentrations, volumes, and sampling times. Urinary flow rates were calculated from urinary volumes and sampling times. The concentrations of U-Cd, U-Alb, and U-A1M were adjusted for U-Crea and SG to compensate for variations in dilution between the different samples. SG_Standard_ = 1.015 was used in SG adjustment calculations (Suwazono et al. 2005).

*Statistics.* Data analyses were performed on untransformed data using SAS software version 9.1 (SAS Institute Inc., Cary, NC, USA). Associations between variables were assessed within individuals by calculations of Spearman correlation coefficients, *r*_S_, (PROC CORR) or by separate linear regression models (PROC REG) for each participant. Because we hypothesized that physiological variation in protein excretions would affect U-Cd excretion (rather than the opposite), U-Cd was the dependent variable in the regression models. Overall mean correlation coefficients and overall mean regression slopes (regression coefficients; βs) were calculated by averaging the participant-specific sample means, and were tested for significant deviation from zero using the Wilcoxon signed rank-test (PROC UNIVARIATE). Differences in overall mean values between groups were tested using the Wilcoxon rank-sum test (PROC NPAR1WAY). Statistical significance was determined at *p* < 0.05, and two-sided confidence intervals were used. For values < LOD, the LOD divided by the square root of 2 was used in the statistical calculations (Hornung and Reed 1990).

## Results

Mean values of the individual mean concentrations and excretion rates of U-Cd, U-Alb, and U-A1M across participants are shown in Table 1. The only significant difference between men and women was that women had lower U-A1M excretion rates (0.10 mg/hr vs. 0.18 mg/hr, *p* = 0.02) than men.

**Table 1:** Means (ranges) of U-Cd, U-Alb, and U-A1M for study participants.

Biomarker	All (N = 354, n = 30)		Men (N = 180, n = 15)		Women (N = 174,a n = 15)		p-Value sex differenceb
	Mean	Range	Mean	Range	Mean	Range	
Cd
U-Cd (µg/L)	0.12	< LODc–1.1	0.11	< LODc–0.53	0.14	< LODc–1.1	0.77
U-CdCrea (µg/gC)d	0.11	0.01–0.52	0.08	0.01–0.25	0.16	0.02–0.52	0.06
U-CdSG (µg/L)e	0.12	0.01–0.71	0.09	0.01–0.31	0.14	0.02–0.71	0.12
U-Cd/hr (µg/hr)f	0.007	0.0007–0.03	0.007	0.0009–0.02	0.008	0.0007–0.03	0.54
Alb
U-Alb (mg/L)	7.5	< LODc–121	10.5	< LODc–121	4.4	< LODc–33	0.44
U-AlbCrea (mg/gC)d	6.3	1.1–78	7.4	1.2–42	5.3	1.1–78	0.37
U-AlbSG (mg/L)e	6.3	0.28–65	7.9	0.32–65	4.7	0.28–62	1.0
U-Alb/hr (mg/hr)f	0.47	0.02–4.9	0.64	0.08–4.5	0.31	0.02–4.9	0.26
A1M
U-A1M (mg/L)	2.4	< LODc–47	3.1	0.12–47	1.7	< LODc–10	0.12
U-A1MCrea (mg/gC)d	2.0	0.11–31	2.2	0.11–31	1.7	0.22–7.6	0.35
U-A1MSG (mg/L)e	1.9	0.13–32	2.3	0.13–32	1.5	0.13–5.8	0.07
U-A1M/hr (mg/hr)f	0.14	0.002–1.8	0.18	0.01–1.8	0.10	0.002–0.54	0.02
Urinary flow rate (mL/hr)	89.2	8.0–420	84.9	8.0–336	93.6	11–420	0.65
Mean values are calculated as mean of 30 individual means; ranges include all 354 urine samples. aOne woman provided samples over 1 day only (6 samples).bTests of sex difference were performed on 30 individual means (Wilcoxon rank-sum test). cValues < LOD were replaced with LOD/√_2 in calculation. dConcentrations adjusted for U-Crea concentration (mean U-Crea, 1.2 g/L). eConcentrations adjusted for SG (mean SG, 1.017). fExcretion rates.										

**Figure 1:**
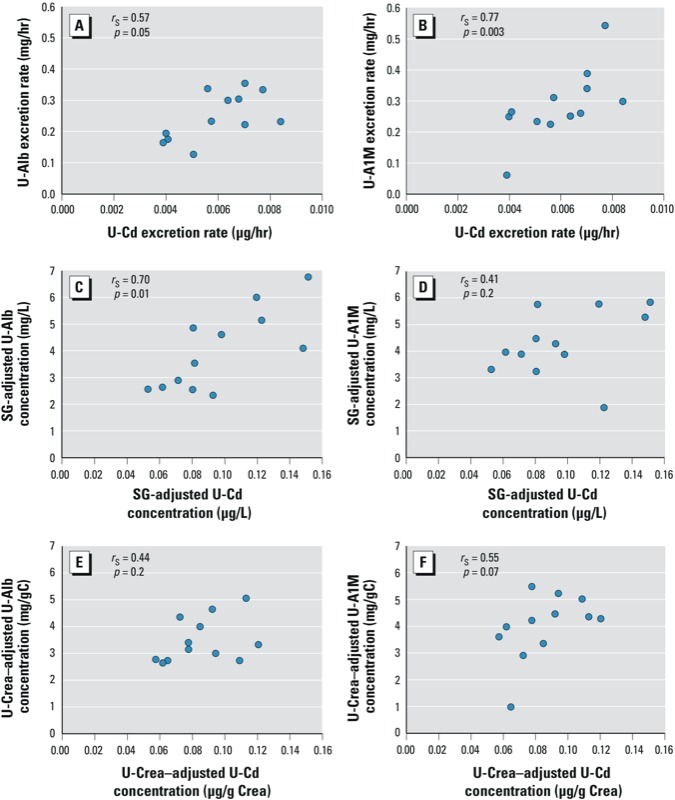
Example showing the association, for 1 of 30 individual participants, between Cd excretion rates (*A*,*B*) and Cd concentrations adjusted for SG (*C*,*D*), and Cd concentrations adjusted for U-Crea concentration (*E*,*F*) and U-Alb (*A*,*C,E*), or U-A1M (*B*,*E*,*F*), respectively.

**Figure 2:**
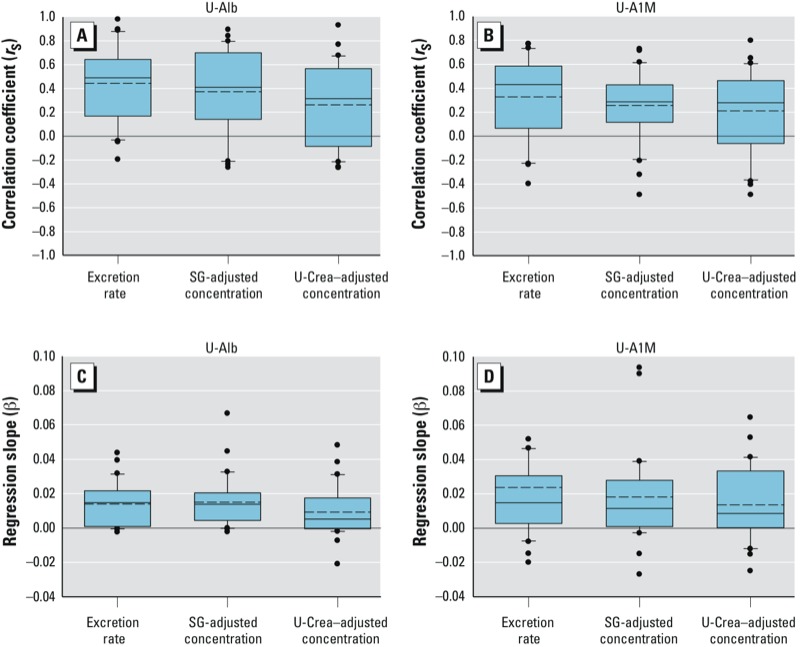
Distributions of individual Spearman correlation coefficients (*r*_S_; *A*,*B*) and individual regression coefficients (β; *C*,*D*) for associations between U-Cd and U-Alb (*A*,*C*), and U-Cd and U-A1M (*B*,*D*), calculated for 30 individual participants [individual values based on 6 samples per day over 2 days for each participant, 354 total samples (1 participant had 6 samples only)]. Boxes indicate 10th and 25th percentiles; dotted line, mean; solid line, median; and whiskers, 75th and 90th percentiles across all participants, with dots indicating outliers. Excretion rates in µg/hr for U-Cd, mg/hr for U-Alb and U-A1M; SG-adjusted concentrations in µg/L for U-Cd, mg/L for U-Alb and U-A1M; U-Crea–adjusted concentrations in µg/g creatinine for U-Cd, mg/g creatinine for U-Alb and U-A1M. All measures deviate significantly from 0 (*p* < 0.05).

*Associations between excretion of U-Cd and U-Alb.* On average, U-Cd excretion was positively associated with U-Alb excretion within individual participants (see Figure 1 for an example of data for all samples from an individual participant and Figure 2 for the distributions of means across participants). The association was stronger for excretion rates (overall mean *r*_S_ = 0.44, *p* < 0.001) and concentrations adjusted for SG (overall mean *r*_S_ = 0.37, *p* < 0.001) compared with concentrations adjusted for U-Crea (overall mean *r*_S_ = 0.26, *p* = 0.001). Exclusion of overnight samples (*n* = 59), values < LOD (*n* = 113), or very diluted or concentrated samples [U-Crea < 0.3 g/L or > 3.0 g/L (*n* = 28), respectively, or SG < 1.010 or > 1.030 (*n* = 85)] did not change the overall results, and there were no significant sex differences in the association between U-Cd and U-Alb (data not shown). Overall means were similar when participants were stratified by age (above/below median 39 years) or BMI (above/below median 23.9 kg/m^2^) (data not shown).

*Associations between excretion of U-Cd and U-A1M.* On average, excretion of U-Cd was positively associated with U-A1M within individual participants (Figures 1 and 2), with stronger overall associations for excretion rates (overall mean *r*_S_ = 0.33, *p* < 0.001) and concentrations adjusted for SG (mean *r*_S_ = 0.26, *p* < 0.001) than for concentrations adjusted for U-Crea (mean *r*_S_ = 0.21, *p* = 0.002). As for U-Alb, overall means for associations between U-A1M and U-Cd were similar after excluding very diluted or very concentrated samples, overnight samples, or samples < LOD (*n* = 92 for U-Cd vs. U-A1M), and there were no significant differences when participants were stratified by age or BMI (data not shown).

Overall mean values of individual linear regression model coefficients were significantly > 0 for U-Alb and U-A1M when both were included in the same model as predictors of U-Cd, regardless of whether the dependent variable was U-Cd excretion rate or U-Cd concentration adjusted for U-Crea or SG (data not shown).

*Associations between urinary flow rates and U-Cd, urinary proteins, and U-Crea.* Regression analyses of data from individual participants indicated significant negative overall mean associations between urinary flow rates and unadjusted concentrations of U-Cd, U-Alb, and U-A1M, consistent with expectations (data not shown). However, mean values for associations between urinary flow and urinary excretion rates across all participants were positive and significantly > 0 for U-Cd, U-Alb, and U-A1M (overall mean β = 0.032 × 10^–3^, β = 1.8 × 10^–3^, and β = 0.49 × 10^–3^, respectively; Table 2). There was also a significant positive overall mean value for the association between urinary flow and U-Crea excretion rates (overall mean *r*_S_ = 0.25, overall mean β = 0.25 × 10^–3^; *p* < 0.001). Overall mean values for associations between urinary flow rates and SG-adjusted concentrations were negative, and they were significantly < 0 for U-Cd and U-Alb. Overall mean values for associations between U-Crea–adjusted concentrations and urinary flow rates were not significant, although nearly so for U-Alb. Similar results were found for mean Spearman correlation coefficients across participants (data not shown). When urinary flow rate was included in regression analyses with U-Cd as the dependent variable and protein excretion (U-Alb or U-A1M) as independent variables, overall mean regression coefficients for urinary flow rate were significantly > 0 for U-Cd excretion and SG-adjusted U-Cd concentration, but not for U-Crea-adjusted U-Cd concentration (Table 3). Overall mean values for associations with U-Alb and U-A1M (Table 3) were similar to overall means from models that were not adjusted for urinary flow rate (Figure 2).

**Table 2:** Mean linear regression model coefficients for associations of urinary flow rate (independent variable mL/hr) with U-Cd, U-Alb, or U-A1M (as dependent variables) based on separate within-individual models for 30 participants.

Adjustment methoda	U-Cd		U-Alb		U-A1M	
	β × 10–3 (p-value)b	Intercept	β × 10–3 (p-value)b	Intercept	β × 10–3 (p-value)b	Intercept
Excretion rate	0.032 (< 0.001)	0.0053	1.8 (< 0.001)	0.32	0.49 (0.02)	0.11
SG adjusted	–0.58 (0.007)	0.14	–15 (0.005)	6.9	–9.0 (0.07)	2.5
U-Crea adjusted	–0.019 (0.49)	0.11	11 (0.05)	5.1	–1.4 (0.30)	2.0
Individual regressions based on data for 6 samples per day over 2 days (354 total samples; one participant had 6 samples only). For each individual participant, X = β(urinary flow rate) + intercept, where X = U-Cd, U-Alb, or U-A1M, respectively. aExcretion rate in µg/hr for U-Cd, mg/hr for U-Alb and U-A1M; SG-adjusted concentrations in µg/L for U-Cd, mg/L for U-Alb and U-A1M; U-Crea–adjusted concentrations in µg/g creatinine for U-Cd, mg/g creatinine for U-Alb and U-A1M. bp-Value for significant deviation of the mean regression coefficient across individuals based on Wilcoxon signed rank test.								

**Table 3:** Mean regression coefficients for the association of U-Cd (dependent variable) with urinary flow (mL/hr) and U-Alb (Model 1) or urinary flow and U-A1M (Model 2), based on separate within-individual models for 30 participants.

Adjustment methoda	Model 1			Model 2		
	β1 (p-value)b	β2 × 10–3 (p-value)b	Intercept	β1 (p-value)b	β2 × 10–3 (p-value)b	Intercept
Excretion rate	0.013 (< 0.001)	0.016 (0.004)	0.0035	0.025 (< 0.001)	0.011 (0.007)	0.0042
SG adjusted	0.014 (< 0.001)	–0.45 (0.02)	0.093	0.019 (< 0.001)	–0.56 (0.02)	0.11
U-Crea adjusted	0.007 (0.007)	–0.096 (0.90)	0.098	0.014 (< 0.001)	–0.089 (0.99)	0.094
Individual regressions based on data for 6 samples per day over 2 days (354 total samples; one participant had 6 samples only). For each individual participant, Model 1: U-Cd = β1(urinary flow rate) + β2 (U-Alb) + intercept; Model 2: U-Cd = β1(urinary flow rate) + β2 (U-A1M) + intercept. aExcretion rate in µg/hr for U-Cd, mg/hr for U-Alb and U-A1M; SG-adjusted concentrations in µg/L for Cd, mg/L for albumin and A1M; U-Crea–adjusted concentrations in µg/g creatinine for Cd, mg/g creatinine for U-Alb and U-A1M. bp-Value for significant deviation of the mean regression coefficient across individuals based on Wilcoxon signed rank test.							

*Estimated effect of U-Crea or SG on U-Cd and proteins.* Overall mean values of within-individual correlation and regression coefficients indicated negative associations between U-Crea and U-Crea-adjusted concentrations of U-Cd, U-Alb, and U-A1M (U-Cd: *r*_S_ = –0.20, *p* = 0.02 and β = –0.02, *p* = 0.07; U-Alb: *r*_S_ = –0.31, *p* < 0.001 and β = –1.8, *p* = 0.006; and U-A1M: *r*_S_ = –0.15, *p* = 0.03 and β = –0.080, *p* < 0.001, respectively) indicating that using U-Crea to adjust for dilution was not perfect. In contrast, SG was not significantly associated with SG-adjusted concentrations of U-Cd, U-Alb, or U-A1M (data not shown).

## Discussion

In this study, with multiple samples from each individual participant, we have estimated associations between U-Cd and urinary proteins used as biomarkers of kidney function. In this way we were able to evaluate whether associations may be caused by factors that vary within individuals and which cannot be differentiated from causal effects of Cd on renal function in studies based on a single spot urine sample from each subject. To the best of our knowledge, this is the first study to evaluate associations between U-Cd and proteins used as biomarkers of kidney function within individuals in a group of environmentally exposed never-smokers.

Our results show clear positive mean associations between the excretion of U-Cd and U-Alb and between excretion of U-Cd and U-A1M at low levels of Cd exposure. Because Cd has a long biological half-life in the human body (EFSA 2009; Järup and Åkesson 2009; Nordberg et al. 2007) the kidney Cd and whole body Cd are expected to be stable during the two sampling days, and urinary Cd should therefore be affected by normal physiological variation only. Thus, the associations between urinary Cd and protein excretion observed within individuals in our study are not consistent with an effect of Cd toxicity.

Chaumont et al. (2012) suggested that protein excretion caused by Cd toxicity is unlikely at low levels of exposure. Instead, these investigators have proposed that Cd-bound metallothionein and certain LMW proteins share the same renal tubular binding site, and that physiological variation in tubular reabsorption will therefore affect excretion of Cd-metallothionein and LMW proteins in the same direction. Removal of proteins such as metallothionein, Alb, and A1M from the tubular fluid by endocytosis is mediated by two multiligand receptors: megalin and cubilin (Christensen et al. 2009). We showed clear associations between urinary flow and U-Cd, U-Alb, and U-A1M, suggesting that variation in urinary flow is an important determinant of variation in tubular reabsorption.

In a study of industrial workers, Haddam et al. (2011) found an association between U-Cd and proteins, but concluded that the association was largely driven by smoking, diuresis, and probably also by a coexcretion of Cd with the proteins. In the present study, U-Alb and U-A1M were significant predictors of U-Cd excretion rate and U-Cd concentration, regardless of which adjustment method for diuresis was used. However, we could not determine whether normal physiological variability in renal tubular reabsorption is the only explanation. Cd in serum is bound not only to the LMW protein metallothionein, but also to Alb (Nordberg 1984). Although Alb is a large protein, it is partly filtered in the glomeruli, and can, by competitive inhibition of tubular reabsorption, also affect LMW proteins such as A1M (Bernard et al. 1987). However, serum Cd is mainly bound to metallothionein in individuals with low-level Cd exposure through ingestion (Nordberg et al. 2007).

Investigating the effect of Cd toxicity on kidney function in a study population with low-level exposure using markers of exposure and effect measured in the same urine sample is difficult, and factors that increase the variability of the biomarkers could either attenuate or overestimate an association. Our results indicate that caution is needed if U-Cd is used as a biomarker of exposure when studying the renal effects of low-level Cd exposure. The fact that diuresis and other sources of normal physiological variability affect U-Cd excretion suggests that U-Cd may be a poor marker of kidney Cd at very low exposure levels. However, although the U-Crea adjustment of Cd in this study was not perfect, U-Crea–adjusted Cd was less affected by normal physiological variations than Cd excretion rate or SG-adjusted Cd concentration.

One limitation of this study is that the participants were recruited from university and hospital departments, but we believe that they were likely to be representative of the nonsmoking healthy general population with respect to normal intraindividual variability in U-Cd and protein excretion. Also, samples were from individuals with very low Cd exposures, and thus it was not possible to assess relations between U-Cd and biomarkers of kidney function at medium or high levels of exposure.

## Conclusions

Our study has shown clear positive overall mean associations between the urinary excretion of Cd and urinary proteins in individuals with low-level exposure to Cd. The overall mean associations were seen both for the LMW protein A1M and the HMW protein albumin. These associations are unlikely to be caused by Cd toxicity, but rather reflect temporary changes in urinary flow or other sources of normal physiological variability that affect the excretion of U-Cd and urinary proteins in the same direction, resulting in an overestimation of the risk of renal toxicity from low-level Cd exposure.
